# Emodin Improves Glucose and Lipid Metabolism Disorders in Obese Mice *via* Activating Brown Adipose Tissue and Inducing Browning of White Adipose Tissue

**DOI:** 10.3389/fendo.2021.618037

**Published:** 2021-05-10

**Authors:** Long Cheng, Shuofeng Zhang, Fei Shang, Yibo Ning, Zhiqi Huang, Runcheng He, Jianning Sun, Shifen Dong

**Affiliations:** ^1^ School of Chinese Materia Medica, Beijing University of Chinese Medicine, Beijing, China; ^2^ Analytical and Testing Center, Beijing University of Chemical Technology, Beijing, China

**Keywords:** emodin, obesity, brown adipose tissue, white adipose tissue, browning of white adipose tissue, lipid metabolic signature

## Abstract

**Background:**

Adipose tissue (e.g. white, brown and brite) plays a critical role in modulating energy metabolism. Activating brown adipose tissue (BAT) and inducing browning in white adipose tissue (WAT) has been proposed to be a potential molecular target for obesity treatment. Emodin is a natural anthraquinone derivative that exhibits variety of pharmacologic effects including lowering lipids and regulating glucose utilization. However, the underlying mechanism of action is still unclear. In the present study, we investigated whether emodin could alleviate obesity *via* promoting browning process in adipose tissue.

**Methods:**

C57BL/6J mice were fed with high fat diet to induce obesity. Emodin at the doses of 40 and 80 mg/kg were orally given to obesity mice for consecutive 6 weeks. Parameters including fasting blood glucose, oral glucose tolerance, blood lipids, and the ratios of subcutaneous white adipose tissue (scWAT) or BAT mass to body weight, and morphology of adipose tissue were observed. Besides, the protein expression of uncoupling protein 1 (UCP1) and prohibitin in BAT and scWAT was determined by immunohistochemistry method. Relative mRNA expression of *Cd137*, transmembrane protein 26 (*Tmem26*) and *Tbx1* in scWAT was analyzed using qRT-PCR. And the protein expression of UCP1, CD36, fatty acid transporter 4 (FATP4), peroxisome proliferator-activated receptor alpha (PPARα) and prohibitin of scWAT and BAT were analyzed using western blotting. In addition, ultra-high-performance liquid chromatography with electrospray ionization tandem mass spectrometry was utilized to detect the small lipid metabolites of scWAT and BAT.

**Results:**

Emodin decreased the body weight and food intake in HFD-induced obesity mice, and it also improved the glucose tolerance and reduced the blood lipids. Emodin treatment induced beiging of WAT, and more multilocular lipid droplets were found in scWAT. Also, emodin significantly increased markers of beige adipocytes, e.g. *Cd137*, *Tmem26* and *Tbx1* mRNA in scWAT, and UCP1, CD36, FATP4, PPARα and prohibitin protein expression in scWAT and BAT. Furthermore, emodin perturbed the lipidomic profiles in scWAT and BAT of obese mice. Emodin increased total ceramides (Cers), lysophosphatidylcholines (LPCs), lyso-phosphatidylcholines oxygen (LPCs-O), and phosphatidylethanolamines oxygen (PEs-O) species concentration in scWAT. Specifically, emodin significantly up-regulated levels of Cer (34:1), LPC (18:2), LPC-(O-20:2), PC (O-40:7), PE (O-36:3), PE (O-38:6), PE (O-40:6), and sphingolipid (41:0) [SM (41:0)], and down-regulated PC (O-38:0), PE (O-40:4), PE (O-40:5) in scWAT of obesity mice. In terms of lipid matabolites of BAT, the emodin remarkably increased the total PCs levels, which was driven by significant increase of PC (30:0), PC (32:1), PC (32:2), PC (33:4) and PC (38:0) species. In addition, it also increased species of LPCs, e.g. LPC (20:0), LPC (20:1), LPC (22:0), LPC (22:1), LPC (24:0), and LPC (24:1). Especially, emodin treatment could reverse the ratio of PC/PE in HFD-induced obese mice.

**Conclusions:**

These results indicated that emodin could ameliorate adiposity and improve metabolic disorders in obese mice. Also, emodin could promote browning in scWAT and activate the BAT activities. In addition, emodin treatment-induced changes to the scWAT and BAT lipidome were highly specific to certain molecular lipid species, indicating that changes in tissue lipid content reflects selective remodeling in scWAT and BAT of both glycerophospholipids and sphingolipids in response to emodin treatment.

## Background

White adipose tissue (WAT) and brown adipose tissue (BAT) play a critical role in modulating energy metabolism (1). The adipocytes within WAT store large amounts of triglycerides as chemical energy in unilocular droplets, which are released into circulation as needed. WAT also functions to produce hormones and cytokines, regulates immune system and supports local tissue frame (2). Increases in WAT mass are directly associated with increased rates of metabolic diseases such as obesity and type 2 diabetes (3). BAT is specialized for energy expenditure, which is characterized by small multi-atrial lipid droplets, abundant mitochondria and expresses uncoupling protein 1 (UCP1) (4, 5). BAT dissipates energy as heat and stores energy for use of non-shivering thermogenesis, which also plays a significant role in energy regulation (5, 6). It has been confirmed that when the body is stimulated by cold exposure (7) or activated by β-adrenoceptors agonist (8), brown-like phenotypic adipocytes (e.g. beige adipocytes) can be detected in WAT, which are characterized by an increased number of mitochondria and increased expression of brown fat marker genes (e.g. *Ucp1*, *Pgc-1α*, *Prdm16*). The above process is called browning of WAT. Recent studies showed that increasing metabolic activity of brown and beige adipose tissue might be a novel way to ameliorate glucose and lipid metabolism in obese patients (9–12). Furthermore, changes in tissue lipid content reflects selective remodeling in scWAT and BAT of both phospholipids and glycerol lipids in response to specific conditions such as exercise training (1).

Emodin (1,3,8-trihydroxy-6-methylanthraquinone) is a natural anthraquinone derivative, and is the main component of *Rheum palmatum* L (13). It has been reported to exhibit anti-inflammatory, anti-bacterial, anti-cancer, anti-diabetic, anti-ulcerogenic, immunosuppressive, pro-apoptotic and chemopreventive activities (14–18). It has been found that the emodin can regulate glucose utilization and lower lipids in epididymal WAT by activating AMP activated protein kinase (AMPK) pathway (19, 20). In addition, the emodin could inhibit adipocyte differentiation and enhances osteoblast differentiation from bone marrow mesenchymal stem cells (BMSCs) (21). And it could improve the inactive glucocorticoid-induced adipose tissue dysfunction by selective inhibition on 11β-hydroxysteroid dehydrogenase type 1 (11β-HSD1) in 3T3-L1 adipocyte (22). Our previous research also showed that emodin could inhibit the accumulation of white adipocytes and inducing browning of WAT in apolipoprotein E knockout (ApoE^-/-^) mice (23). However, the effect of emodin on the lipid metabolic signature of scWAT and BAT has not been investigated. Here, we report the effect of emodin on adipose tissue in high fat diet-induced obese mice, as well as a comprehensive analysis of lipid composition in scWAT and BAT.

## Materials and Methods

### Chemicals and Reagents

Emodin (purity 95%) was purchased from Shanghai Yuanye Biotechnology Co., Ltd. CL 316243 disodium salt was purchased from APExBIO Technology LLC (Houston, USA). Biochemical kits of serum total cholesterol (TC), triglyceride (TG), high-density lipoprotein cholesterol (HDL-c), and low-density lipoprotein cholesterol (LDL-c) were purchased from Nanjing Jiancheng Bioengineering Institute (Nanjing, China). Free fatty acid (FFA) ELISA assay kit was purchased from Jiangsu Kete Biotechnology Co., Ltd. (Jiangsu, China). Leptin ELISA assay kit and adiponectin ELISA assay kit were purchased from cloud-clone Corp. Wuhan (Wuhan, China).

### Animals and Experimental Protocol

Eight-week-old male C57BL/6J mice weighing 18-22g were purchased from Sibeifu (Beijing) Biotechnology Co., Ltd (grade SPF, Certificate No: SCXK (jing) 2016-0002). Mice were maintained at 23 ± 1°C and 60-70% humidity with a 12h light/dark cycle. The regular diet was a standard chow diet containing 3.65 kcal/g. And the high fat diet was 60% of kilocalories from fat containing 5.24 kcal/g. Normal diet is purchased from SBF (Beijing) Biotechnology Co., Ltd (Beijing, China, 0817SH08200438C). High fat diet was purchased from Beijing huafukang Biotechnology Co., Ltd (Beijing, China, 20180376).

Mice were randomly divided into two groups according to weight and fed with normal control diet (*n*=8) or fed with high fat diet (HFD) for 8 weeks to induce hyperlipidemia. After 8 weeks of HFD, mice were randomly divided into four groups as follows (*n*=8/group): HFD group, emodin 40 mg/kg group, and emodin 80 mg/kg group, and CL 316243 1 mg/kg group. Mice in normal control group and HFD group were administrated with equal amount of 0.1% carboxymethyl cellulose-Na (CMC-Na). Mice in emodin 40 and 80 mg/kg groups were taken emodin (dissolved in 0.1% CMC-Na) by intragastric administration for consecutive 6 weeks. Before dissection, mice in CL 316243 1 mg/kg treatment group were intraperitoneally injected with 1mg/kg/day of CL 316243 disodium salt for 3 days. All the animal studies were in accordance with ethics standards of the Animal Care and Welfare Committee of Beijing University of Chinese Medicine (Certificate No. BUCM-04-2018070603-3015).

### Oral Glucose Tolerance Test

After 6 weeks of intervention, the mice were fed by oral gavage with 50% D-glucose (2.0 g/kg) after overnight (12 h) fasting. Blood samples were taken from the tail 0, 30, 60, 90 and 120 min after oral gavage, and glucose levels were measured by the One Touch Ultra blood glucose monitoring system (ONETOUCH Ultra Easy).

### Measurement of Lee’s Index

At the end of the treatment, the body mass of the mice was accurately weighed, and the body length (the distance from the tip of the nose to the anus) was accurately measured, and then the Lee’s index was calculated according to the reference (24).

### Measurement of Adipose Tissue Mass/Body Weights

Subcutaneous WAT (scWAT) mass and scapular brown adipose tissues (BAT) mass were accurately weighed. The ratios of scWAT mass to body weight (BW) and BAT mass to body weight (BW) were calculated.

### Serum Biochemical Analysis

Serum TC, TG, HDL-c and LDL-c levels were measured with the method of biochemical kits (Nanjing Jiancheng, China). The levels of FFA were determined by the Mouse FFA ELISA kit (Kete, China). Serum leptin and adiponectin levels were analyzed using the mouse leptin and adiponectin ELISA kit respectively (Cloud-clone, China).

### Histological and Immunohistochemical Analysis

BAT and scWAT were fixed with 10% formalin, dehydrated, embedded in paraffin and sectioned. For histological analysis, sections were deparaffinized and stained with hematoxylin and eosin. The expression of UCP1 (1:500, ab10983, Abcam) and prohibitin (1:500, ab75766, Abcam) in mouse BAT and scWAT was determined by immunohistochemistry. All images were acquired with the microscope (Leica, Germany). The expression level of UCP1 and prohibitin in BAT and scWAT of the mice was quantified by using Image Pro Plus 6.0.

### Quantitative Real-Time PCR Analysis

Total RNA of scWAT was extracted with Trizol^®^ Reagent (Ambion, USA). Reverse transcription of total RNA (1 μg) was performed with Revert Aid First Stand cDNA Synthesis Kit (Thermo Scientific, USA). Real-time quantitative PCR (qRT-PCR) was performed with a SYBR Green Master Mix (Novoprotein, China). The PCR reaction was operated in triplicate for each sample using the Step One Real-Time PCR System (Applied Biosystems, USA). After standardizing the expression level of internal control actin in each sample, the data were expressed in arbitrary units. The sequences of primer in this study were shown in **Table 1**.

**Table 1:** Primer sequences were used for quantitative real-time reverse transcription polymerase chain reaction (qRT-PCR).

Gene name	Forward (5’-3’)	Reverse (5’-3’)
*Cd137*	GGTGGACAGCCGAACTGTAA	GCTGCTCCAGTGGTCTTCTT
*Tmem26*	AGTGTGAGCAAGAACTCGGG	GATGGCCGGAGAAAGCCATT
*Tbx1*	CGCTACCGGTATGCTTTCCA	GTCTTTTCGAGGGGCCACAT
*β-actin*	GGTGGGAATGGGTCAGAAGG	GTTGGCCTTAGGGTTCAGGG

### Western Blot Analysis

The homogenates of scWAT and BAT were dissolved in RIPA lysate and protease inhibition for protein extraction. Sample protein concentrations were measured using the bicinchoninic acid (BCA) method (Beyotime). Total protein (10μg/Lane) was separated on a 12% acrylamide/acrylamide gel using sodium dodecyl sulfate-polyacrylamide gel electrophoresis (SDS-PAGE) and transferred to polyvinylidene difluoride (PVDF) membranes. PVDF membranes containing protein were incubated with specific anti-alpha tubulin antibody (1:5000, ab18251, Abcam), anti-UCP1 antibody (1:1000, ab10983, Abcam), anti-Prohibitin antibody (1:10000, ab75766, Abcam), anti-PPAR α antibody (1:2000, ab8934, Abcam), anti-CD36 antibody (1:5000, ab133625, Abcam), anti-slc27a4/FATP4 antibody (1:1000, ab200353, Abcam), respectively. Then membranes were incubated with HRP-conjugated Affinipure Goat Anti-Mouse IgG (H+L) (1:5000, 20000175, proteintech) or HRP-conjugated Affinipure Goat Anti-Rabbit IgG (H+L) (1:5000, 20000174, proteintech). Protein bands were visualized using the ECL kit (EMD millipore). Image was analyzed using Image-Pro-Plus 6.0.

### Targeted Lipidomics Analysis

#### Tissue Sample Preparation

BAT and scWAT tissue samples were thawed on ice. Samples were accurately weighed and then homogenized in the 1.5 mL centrifuge tube using a Speed Mill Plus. Internal standards were dissolved in 300μL of methanol [SPLASH^®^ II LIPIDOMIX^®^ Mass Spec Standard (330709), Cer/Sph Mixture I (LM6002, Avanti), 12:0-13:0 PC (LM1000, Avanti), 12:0-13:0 PE (LM1100, Avanti)] and added to each sample, and then extracted with 1mL of methyl tert-butyl ether (MTBE) for 1 hour. The extraction was added 250 μL of water and pelleted in a 4°C centrifuge at 12,000 rpm for 5 min. 100 μL of the MTBE layer was transferred to a new 1.5 mL centrifuge tube and dried in a Savant™ SpeedVac™ High Capacity Concentrator. The dried sample was reconstituted with 400 μL of isopropanol/acetonitrile (1:1) and shaken for 40s. And the dissolved matter was centrifuged at 12,000 for 5 min, and then 100 μL of the supernatant was transferred to a 200 μL vial insert for liquid chromatography-mass spectrometry analysis.

### Chromatography

ACQUITY Ultra Performance Liquid Chromatography (UPLC) I-Class System (Waters, USA) with ACQUITY UPLC BEH C_8_ Column (2.1 mm×100 mm, 1.7 μm) was used to perform the UPLC separation. For C_8_ separation, mobile phase A is acetonitrile/water (60/40) and mobile phase B is acetonitrile/isopropanol (90/10), and both A and B contain 0.1% formic acid and 5 mM ammonium acetate (formate). The gradient conditions were shown in **Tables 2** and **3**.

**Table 2:** The gradient conditions for reversed phase C_8_ separation for lipids.

Time(min)	A (v%)	B (v%)
0	68	32
1.5	68	32
15.5	15	85
15.6	3	97
18	3	97
18.1	68	32
20	68	32

**Table 3:** The gradient conditions for reversed phase C_8_ separation for fatty acids.

Time(min)	A(*v%*)	B(*v%*)
0	90	10
1.5	90	10
8	3	97
13	3	97
13.1	90	10
15	90	10

### Quality Control

Five QC samples of adipose tissue were continuously injected at the beginning of the sequence to monitor the UPLC-MS system stability by the Overlay Graphs method using Mass Lynx software. And QC samples were run at regular intervals (8 samples) throughout the entire sequence.

### Mass Spectrometry

Electrospray ionization tandem mass spectrometry (XEVO TQ-S Micro, Waters, USA) was used for mass spectrometry. And the conduct conditions of ESI^+^ and ESI^-^ showed in **Table 4**. Masslyxn4.1 was used for mass spectrometry data acquisition.

**Table 4:** Analysis condition of positive and negative electrospray ionization.

Parameter	ESI^+^	ESI^-^
Capillary voltage	3200V	2000V
Desolvation temperature	500°C	500℃
Source temperature	120°C	120℃
Desolvation gas flow	1000 L/h	1000L/h
Cone gas flow	150 L/h	150 L/h
Nebuliser gas	7.0 bar	7.0 bar
Collision gas flow	0.13 L/h	0.13 L/h

### Statistical Analysis

The data of target metabolism group were operated by skyline 19.1. The parameters were set as follows: the quality extraction error was 5 ppm and the allowable retention time error was 15s. Other data were statistically analyzed using SAS 8.2 software. All data were expressed as means ± SE. Two-way analysis of variance for repeated measures was used for body weight analysis (for the effects of treatment and time). Other statistics was performed using the one-way analysis of variance (ANOVA) followed by SNK-*q* test. *P*-value<0.05 was considered as statistically significant.

## Results

### Emodin Can Inhibit Obesity and Appetite and Reduce Fat Mass in HFD Induced Obese Mice

**Figure 1:**
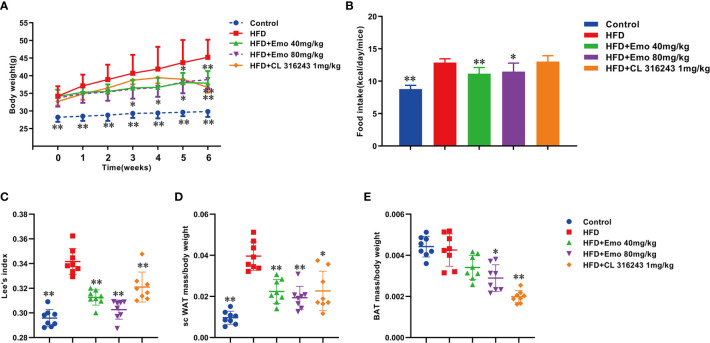
Effects of emodin on body weight, food intake and Lee’s index in HFD mice. Mice were fed with high fat diet (HFD) containing 5.24 kcal/g for 8 weeks to induce hyperlipidemia. The mice in HFD+Emo group were intragastrically administered with emodin at the doses of 40 and 80 mg/kg/day respectively for consecutive 6 weeks. The mice in HFD+CL 316243 group were intraperitoneally injected with 1mg/kg/day of CL 316243 disodium salt for 3 days just before detecting time point. **(A)** Body weight. **(B)** Food intake. **(C)** Lee’s index. **(D)** The ratio of scWAT mass/BW. **(E)** The ratio of BAT mass/BW. HFD, high fat diet; Emo, emodin. Data are expressed as mean ± SE, with *n* = 8. ^*^ *P* < 0.05, ^**^ *P* < 0.01 *vs.* HFD group.

The HFD treated mice showed characteristics of obesity. When compared with the control mice, parameters including body weight, food intake, Lee’s index and scWAT/BW ratio were significantly increased in HFD treated mice (by 51.6%, 41.1%, 8.5% and 39.1%, respectively). Emodin at the dose of 40 mg/kg caused a significant reduction in body weight at week 5 and 6 (by 13.0% and 15.7%, respectively), and emodin 80 mg/kg caused a remarkable reduction in body weight at week 3, 4, 5, 6 (by 11.1%, 12.6%, 12.6% and 13.9%, respectively), when compared with HFD mice (**Figure 1A**).

Emodin (40 mg/kg, 80 mg/kg) significantly decreased the food intake by 9.8% and 7.3%, respectively, when compared with the obese mice (*P <*0.01 or *P <*0.05) (**Figure 1B**).

Lee’s index can be used as an indicator to evaluate the degree of obesity in adult obese model mice (25). Emodin at the doses of 40 and 80 mg/kg and CL316243 (1 mg/kg) treatment group could significantly reduce the Lee’s index, when compared with HFD mice (*P <*0.01) (**Figure 1C**).

The ratio of scWAT to BW in mice treated with emodin (40mg/kg, 80mg/kg) and CL316243 (1mg/kg) was significantly decreased (by 39.1%, 46.4% and 40.9%, respectively), when compared with the HFD mice (P < 0.01 or P < 0.05). (**Figure 1D**).

The function of BAT is consuming glucose and lipids, mediating the thermogenic effects of non-shivering, thereby increasing energy expenditure (26). Interestingly, compared with HFD mice, emodin (80 mg/kg) and CL 316243 (1 mg/kg) treatment significantly decreased the ratio of BAT/BW (by 22.5% and 47.5%, respectively (**Figure 1E**).

### Emodin Ameliorates Abnormal Blood Glucose and Blood Lipid in Mice Fed With HFD

**Figure 2:**
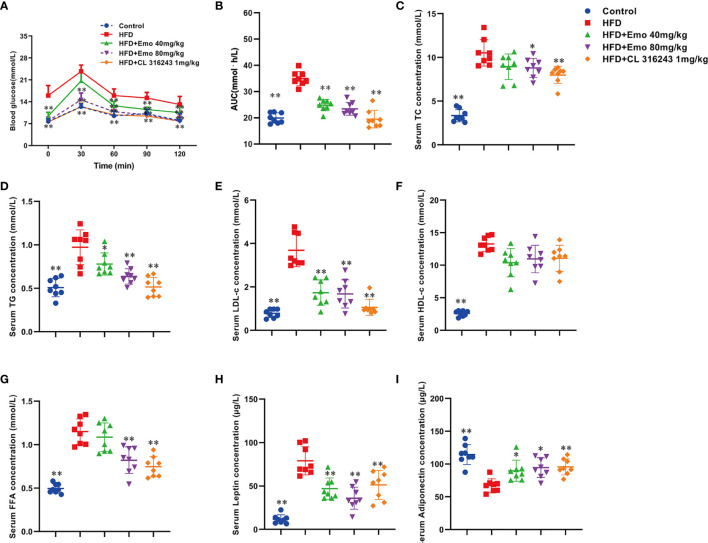
Effects of emodin on blood glucose and lipids in HFD mice. **(A)** Oral glucose tolerance test (OGTT). The mice were fasted for 12 h, and then 2 g/kg glucose was given oral gavage. Glucose levels were tested before (0) and after fed glucose at 30, 60, 90, and 120 min. **(B)** Quantification of AUC from the OGTT. **(C–F)** Serum TC, TG, LDL-c, HDL-c concentration. **(G)** Serum free fatty acids concentration. **(H)** Serum leptin concentration. **(I)** Serum adiponectin concentration. HFD, high fat diet; Emo, emodin. Data are expressed as mean ± SE, with n = 8. ^*^ *P* < 0.05, ^**^ *P* < 0.01 *vs.* HFD group.

In this study, we investigated whether emodin improved glucose tolerance in obese mice. The results indicated an impaired glucose tolerance in HFD mice, and high fat diet significantly increased AUC index, when compared with control mice (*P <*0.01). Emodin at the doses of 40 and 80 mg/kg and CL316243 (1 mg/kg) treatment significantly decreased AUC value (31.1%, 35.3% and 45.1% respectively), when compared with HFD mice (*P <*0.01) (**Figures 2A, B**). These results suggested that emodin could ameliorate glucose metabolism in obese mice.

To investigate whether emodin improved hyperlipidemia in obese mice, blood lipid parameters were measured. When compared with control mice, serum TC, TG, LDL-c, HDL-c and FFA levels were significantly increased in obese mice (*P <*0.01). When compared with obese mice, emodin (40 mg/kg, 80 mg/kg) could remarkably decrease serum TC, TG and LDL-c by 15.1%-16.3%, 19.6%-34.0%, 52.9%-54.3%, respectively (*P <*0.01 or *P <*0.05) (**Figures 2C–E**), and emodin (80 mg/kg) could remarkably decrease serum FFA levels (**Figure 2G**), but there was no significant difference in the content of HDL in serum (**Figure 2F**).

Leptin plays an important role in maintaining energy metabolism and regulating adipose ratio (27). It was demonstrated that the serum leptin content of HFD mice was significantly increased by 574.3%, when compared with control mice (*P <*0.01). Emodin (40 mg/kg, 80 mg/kg) and CL316243 (1 mg/kg) caused significant reduction in leptin levels (by 40.7%, 54.6% and 41.5%, respectively), when compared with HFD mice (*P <*0.01) (**Figure 2H**).

As an endogenous insulin sensitizer secreted by adipose tissue, reduction of adiponectin is an independent risk factor for hyperlipidemia and diabetes (28). When compared with control mice, serum adiponectin in HFD mice was significantly decreased (*P <*0.01). Emodin (40 mg/kg, 80 mg/kg) treatment could significantly increase the serum adiponectin levels in HFD mice (by 28.6% and 42.9%, respectively), when compared with HFD mice (*P <*0.01 or *P <*0.05) (**Figure 2I**).

### Emodin Induces Browning of scWAT in Mice Fed With HFD

**Figure 3:**
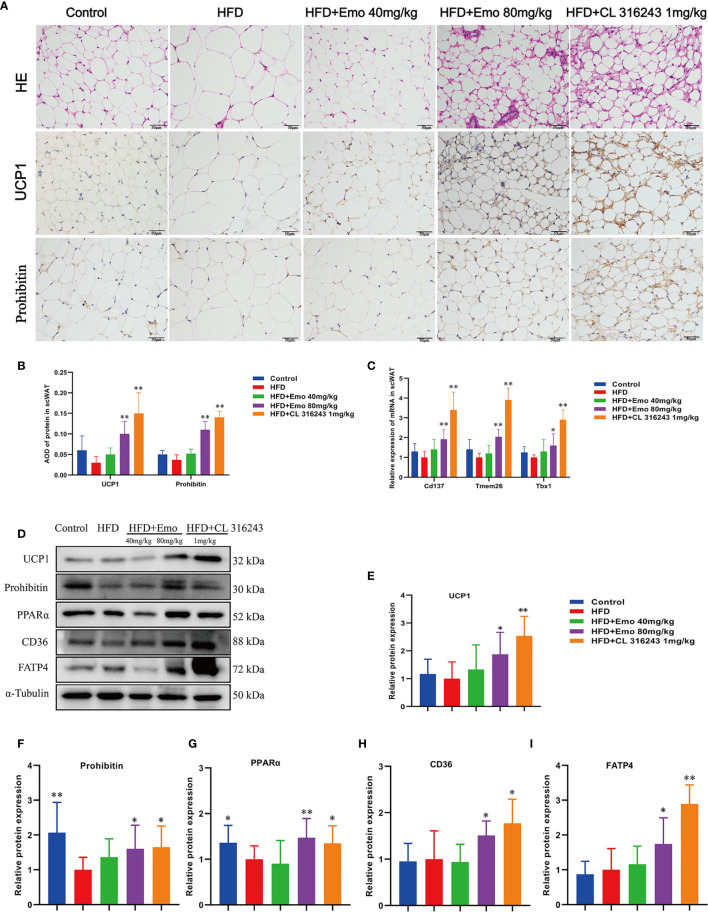
Effects of emodin on the morphology and function of scWAT in HFD mice. **(A)** H&E and immunohistochemical staining of scWAT. **(B)** Relative expression of UCP1 and Prohibitin protein in scWAT. **(C)** Relative expression of *Cd137*, *Tmem26* and *Tbx1* mRNA in scWAT. **(D)** Protein expression of UCP1, prohibitin, PPAR α, CD36 and FATP4 in scWAT using western blotting. **(E)** Protein expression of UCP1. **(F)** Protein expression of prohibitin. **(G)** Protein expression of PPAR α. **(H)** Protein expression of CD36. **(I)** Protein expression of FATP4. HFD, high fat diet; Emo, emodin. Data are expressed as mean ± SE. ^*^ *P* < 0.05, ^**^ *P* < 0.01 *vs.* HFD group.

We analyzed the morphology of scWAT and the expression of thermogenic protein UCP1 and mitochondrial membrane protein prohibitin in scWAT (**Figure 3A**). When compared with the control mice, the diameter of fat cells in HFD mice increased and the number of cells per unit area decreased. When compared with HFD mice, the adipocytes of the mice in the emodin (40 mg/kg, 80 mg/kg) groups are small and tightly arranged, with obvious nuclei. As an important thermogenic protein, UCP1 is specifically expressed in BAT (6, 29). Prohibitin, mainly located in the inner membrane of mitochondria, plays an important role in maintaining mitochondrial morphology, function and regulating energy metabolism (30). Therefore, we measured the expression of UCP1 and prohibitin protein in scWAT (**Figure 3B**). The expression of UCP1 and prohibitin protein in scWAT of emodin 80 mg/kg-treated group was significantly increased (*P <*0.01), Compared with HFD mice. We also evaluated the mRNA expression of beige adipocyte marker genes, such as *Cd137*, Transmembrane protein 26 (*Tmem26*) and *Tbx1*. As expected, the expression of several beige adipocyte marker genes, including *Cd137*, *Tmem26* and *Tbx1*, was significantly upregulated in scWAT after emodin (80 mg/kg) and CL316243 (1 mg/kg) treatment (**Figure 3C**).

In order to confirm whether emodin can induce the browning of scWAT, we measured thermogenic protein and fatty acid transporter (**Figure 3D**). The expression of UCP1, prohibitin, CD36, FATP4 and PPARα protein in scWAT of emodin (80 mg/kg) and CL316243 (1 mg/kg) treatment group increased significantly, when compared with HFD mice (*P <*0.05 or *P <*0.01) (**Figures 3E–I**). These results suggested that emodin could induce the browning of scWAT in HFD mice.

### Emodin Activates Brown Adipose Tissue in Mice Fed With HFD

**Figure 4:**
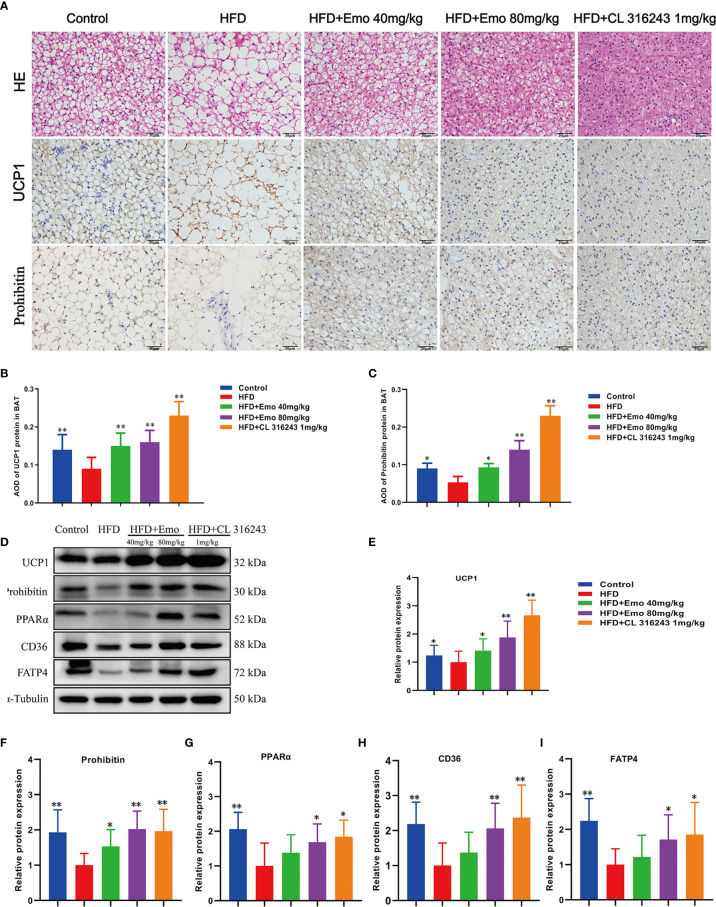
Effects of emodin on the morphology and function of BAT in HFD mice. **(A)** H&E and immunohistochemical staining of BAT. **(B)** Relative expression of UCP1 in BAT. **(C)** Relative expression of prohibitin in BAT. **(D)** Protein expression of UCP1, prohibitin, PPAR α, CD36 and FATP4 in BAT using western blotting. **(E)** Protein expression of UCP1. **(F)** Protein expression of prohibitin. **(G)** Protein expression of PPAR α. **(H)** Protein expression of CD36. **(I)** Protein expression of FATP4. HFD, high fat diet; Emo, emodin. Data are expressed as mean ± SE. ^*^ *P* < 0.05, ^**^ *P* < 0.01 *vs.* HFD group.

We also analyzed the morphology of BAT and the expression of thermogenic protein UCP1 and mitochondrial membrane protein prohibitin in BAT (**Figures 4A–C**). When compared with control mice, the diameter of fat cells in BAT of HFD mice was significantly increased, the number of cells per unit area decreased, and the number of white fat cells increased. This indicates that long-term HFD feeding results in so-called ‘whitening’ of BAT. When compared with HFD mice, the adipocytes of the mice in the emodin (40 mg/kg, 80 mg/kg) groups are small and tightly arranged, with large and obvious nuclei. We also measured the expression of UCP1 and prolibitin protein in BAT. The expression of UCP1 and prohibitin protein in BAT of emodin and CL316243-treated group was significantly increased (*P <*0.05 or *P <*0.01), when compared with HFD mice.

To confirm that emodin can activate BAT of HFD mice, we measured thermogenic protein and fatty acid transporter (**Figure 4D**). The expression of UCP1, prohibitin, CD36, FATP4 and PPARα protein in BAT of emodin and CL316243-treated group increased significantly, when compared with HFD mice (*P <*0.05 or *P <*0.01) (**Figures 4E–I**). These results suggested that emodin could activate BAT in HFD mice.

### Phospholipid Metabolism Is Altered in scWAT With Emodin Treatment

**Figure 5:**
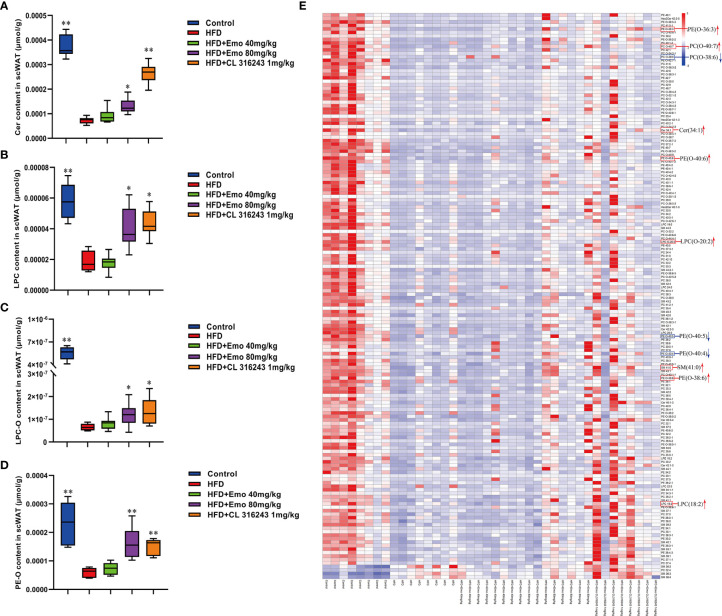
Effects of emodin on the lipid metabolites in scWAT of HFD mice. **(A)** The content of Cer in scWAT. **(B)** The content of LPC in scWAT. **(C)** The content of LPC-O in scWAT. **(D)** The content of PE-O in scWAT. **(E)** Heat map. Only metabolites with VIP >1 and *P <*0.05 were selected in heat map, and different shades of color present the concentration (red, white and blue presented the high, normal and low concentration). The red up arrow indicates up regulation or promotion, and the blue down arrow indicates down regulation or inhibition. HFD, high fat diet; Emo, emodin; PC, phosphatidylcholine; PE, phosphatidylethanolamine; Cer, ceramides; LPC, lyso-phosphatidylcholine. Data are expressed as mean ± SE. ^*^ *P* < 0.05, ^**^ *P* < 0.01 *vs.* HFD group.

Based on the above experimental results, we analyzed scWAT by targeted metabolomics. We selected the biomarkers that have changed and created a heat map (**Figures 5A–E**). The lipidomics data show that emodin treatment can perturb the lipidomics profile in HFD mice, and several phospholipid species (e.g. Cer, LPC, LPC-O, and PE-O) are remarkably increased in scWAT, indicating a remodeling of phospholipids after emodin 80 mg/kg treatment. Specifically, when compared with HFD mice, concentration of Cer (34:2), LPC (18:2), LPC-(O-20:2), PC (O-40:7), PE (O-36:3), PE (O-38:6), PE (O-40:6), and SM (41:0) was significantly up-regulated in emodin-treated group. Otherwise, levels of PC (O-38:6), PE (O-40:4), PE (O-40:5) were significantly reduced in emodin-treated group, when compared with HFD mice.

### Phospholipid Metabolism Is Altered in BAT With Emodin Treatment

**Figure 6:**
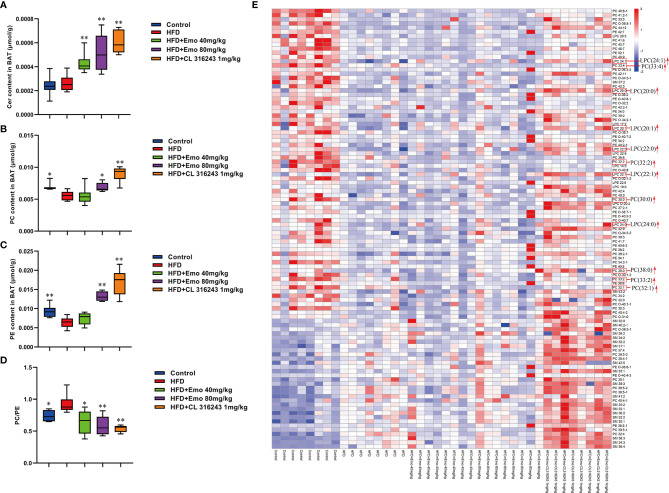
Effects of emodin on the lipid metabolites in BAT of HFD mice. **(A)** The content of Cer in BAT. **(B)** The content of PC in BAT. **(C)** The content of PE in BAT. **(D)** The ratio of PC : PE in BAT. **(E)** Heat map. Only metabolites with VIP >1 and *P <*0.05 were selected in heat map, and different shades of color present the concentration (red, white and blue presented the high, normal and low concentration). The red up arrow indicates up regulation or promotion. HFD, high fat diet; Emo, emodin; PC, Phosphatidylcholine; PE, phosphatidylethanolamine; Cer, ceramides. Data are expressed as mean ± SE. ^*^ *P* < 0.05, ^**^ *P* < 0.01 *vs.* HFD group.

In addition, we also analyzed BAT by targeted metabolomics. We selected the biomarkers that have changed and created a heat map (**Figures 6A–E**). In BAT, the lipidomics indicated a significant reduction of PEs and PCs in BAT of HFD mice. And emodin treatment caused a significant increase of PEs and PCs in BAT, when compared with HFD mice. The increase in total PC was driven by significant increase of PC (30:0), PC (32:1), PC (32:2), PC (33:4) and PC (38:0) species, as well as species of LPC [e.g. LPC (20:0), LPC (20:1), LPC (22:0), LPC (22:1), LPC (24:0), LPC (24:1)] with emodin treatment. In addition, when compared with the control mice, the ratio of PC/PE was significantly increased in HFD-induced mice (*P <*0.05 or *P <*0.01). And emodin at the doses of 40 and 80 mg/kg and CL 316243 significantly decreased the ratio of PC/PE compared with HFD-induced mice.

## Discussion

Emodin ameliorates adiposity and improves whole-body metabolic balance in obese mice. In this study, we demonstrated that emodin could decrease the body weight and food intake in high fat diet induced obese mice, also it could improve the glucose tolerance and reduce the blood lipids, which was consistent with the results of previous studies *in vivo* (22, 31, 32). The regulating of white adipose tissue and brown adipose tissue acts a critical role in combating obesity and metabolic disease. As a thermogenic tissue, BAT is innervated by both sympathetic and sensory nerves. The activity and metabolism of BAT could be influenced by cold exposure and exercise (33), as well as some natural product extracts or compounds, such as rose hip supplement (34), black raspberry (35), green tea extract (36), genistein (37), pentamethylquercetin (38), and etc. Here, we first proved that emodin could promote browning in scWAT. The white adipose tissue mass was decreased after emodin treatment. After treatment of emodin, more multilocular lipid droplets were found in scWAT, the mRNA expression of browning markers including *Cd137*, *Tmem26* and *Tbx1* was increased in scWAT, and the protein expression of browning markers including UCP1 and prohibitin was increased in scWAT of obese mice. Meanwhile, the protein expression of UCP1, prohibitin, PPARα was increased in BAT of obese mice after emodin treatment. PPAR α is the key factor of BAT thermogenesis, which can regulate lipid catabolism and thermogenic gene expression in coordination with *Pgc-1α* and *Prdm16* (39). PPAR α can enhance the expression of PGC-1α and UCP-1 by increasing the activity of erythropoietin (EPO). PPAR α also plays a coordinating role with SIRT1 activated by EPO and jointly regulates the level of NAD^+^ to heighten the metabolic activity (40). The mitochondria are involved in the metabolic control of brown adipocytes. Mitochondrial function is related to the endocrine function of adipocytes. In addition, brown adipocytes rely on mitochondria to maintain intracellular metabolism. Located in mitochondrial inner membrane, prohibitin plays a critical role in maintaining the shape and function of mitochondria and regulating energy metabolism (30, 41). The results of western blotting demonstrated that emodin increased the protein expression of PPAR α and prohibtin of scWAT and BAT in obese mice. As a fatty acid translocase, CD36 acts a pivotal part in the uptake and transport of long-chain fatty acids (LCFA) in heart and adipose tissues (42, 43). It was found that cold exposure drastically accelerated plasma clearance of triglycerides as a result of increased uptake into BAT, a process crucially dependent on local LPL activity and transmembrane receptor CD36 (44). Fatty acid transporter 4 (FATP 4) is a member of the fatty acid transport proteins (FATPs), which plays a significant role in the transport of long-chain fatty acids with more effectively compared with FATP1. It was found that FAT/CD36 and FATP4 were the most effective fatty acid transporters (45, 46). In this study, emodin accelerated the transport and consumption of fatty acids and improved the disorder of lipid metabolism by increasing the expression of CD36 and FATP4 protein in both scWAT and BAT of HFD-induced mice.

White and brown adipocytes exhibit different lipid metabolic signature, which reflect their distinct organelle composition and cell functions. The neutral lipids in the lipid droplets core are surrounded by a monolayer of phospholipids (47). PCs, PEs and CLs make up 89% of the phospholipids in BAT, which are increased in response to cold exposure and exercise (48). It was confirmed that regulation and metabolism of PCs, PSs and PEs prevented inflammation of adipose tissue, hyperlipidemia and obesity (49). Exercise can increase specific molecular species of PCs and PEs in brown adipocytes. It has been reported that after exercise, the increase in total PC was driven by the significant increase of the highly abundant PC (36:2) species, as well as increases in numerous species of PC and PC-O. However, there was no overall change in abundance of PE after exercise in BAT (1). Our recent study manifested that the lipidomic profile of adipocytes was remolded with high fat diets, and emodin treatment could perturb the profile and reverse some small lipid metabolites of HFD mice.

Furthermore, the relative abundance of PCs and PEs on the surface of LDs is important for their dynamics (50). An increase in the relative amount of PEs on the surface of lipid droplets can promote fusion of smaller droplets into larger ones (51). Inhibition of PCs biosynthesis can promote TG storage increases the size of the lipid droplets presumably (52, 53). Either abnormally high, or abnormally low cellular PC : PE molar ratios can influence energy metabolism in various organelles (50). It has been shown that both PC amount and PC : PE molar ratio tend to increase, and palmitate- and stearate-containing LPC species were upregulated in 16-week-old Lep^ob/ob^ adipose tissue macrophage, which related to WAT inflammation and contribute to the development of insulin resistance in obesity (54). It has been proved the different composition of phospholipids in white and brown adipocyte, and thermogenic adipocytes possess higher abundance of PCs and PEs, with longer (C >36) and more polyunsaturated species (55). Our results also indicated that the ratio of PC : PE was significantly increased in brown adipose tissue but not scWAT in HFD-induced mice compared with control mice. Interestingly, emodin 40 and 80 mg/kg treatment and CL 316243 could significantly increase abundance of PCs and PEs, and decrease the PC : PE ratio in BAT of obesity mice. Specifically, emodin significantly up-regulated levels of Cer (34:2), LPC (18:2), LPC-(O-20:2), PC (O-40:7), PE (O-36:3), PE (O-38:6), PE (O-40:6), and SM (41:0), and down-regulated PC (O-38:6), PE (O-40:4), PE (O-40:5) in scWAT compared with HFD mice. And in BAT, the remarkable increase in total PCs was driven by significant increase of PC (30:0), PC (32:1), PC (32:2), PC (33:4) and PC (38:0) species with emodin treatment. In addition, emodin significantly increased species of LPC (e.g. LPC (20:0), LPC (20:1), LPC (22:0), LPC (22:1), LPC (24:0), when compared with HFD mice.

## Conclusion

These results indicated that emodin could ameliorate adiposity and improve metabolic disorders in obese mice. Also, emodin could promote browning in scWAT and activate the BAT activities. In addition, emodin treatment-induced changes to the scWAT and BAT lipidome were highly specific to certain molecular lipid species, indicating that changes in tissue lipid content reflects selective remodeling in scWAT and BAT of both glycerophospholipids and sphingolipids in response to emodin treatment.

## Data Availability Statement

The raw data supporting the conclusions of this article will be made available by the authors, without undue reservation.

## Ethics Statement

All the animal studies were in accordance with ethics standards of the Animal Care and Welfare Committee of Beijing University of Chinese Medicine [Certificate No. BUCM-04-2018070603-3015].

## Author Contributions

JS and SD designed experiments. LC, SZ, FS, YN, ZH, RH, and SD performed experiments. FS and LC performed UPLCQ-MS/MS analysis. LC and SD performed statistical analysis. LC wrote the paper. All authors contributed to the article and approved the submitted version.

## Funding

This paper was supported by the National Natural Science Foundation of China (Grant No. 81503287, 81430094), Natural Science Foundation of Beijing Municipality (Grant No. 7144222, 7174312), and Doctoral Program Foundation of Institutions of Higher Education of China (Grant No. 20130013120002).

## Conflict of Interest

The authors declare that the research was conducted in the absence of any commercial or financial relationships that could be construed as a potential conflict of interest.

## Abbreviations

WAT, white adipose tissue; BAT, brown adipose tissue; HFD, high-fat diet; scWAT, subcutaneous white adipose tissue; UCP1, uncoupling protein 1; PPARα, Peroxisome proliferator-activated receptor alpha; PGC-1α, Peroxisome proliferator activated receptor γ coactivator-1α; PRDM16, positive regulatory domain-containing 16; PHB, prohibitin; AMPK, AMP activated protein kinase; SREBP, sterol regulatory element-binding protein; Tmem26, Transmembrane protein 26; TC, total cholesterol; TG, triglyceride; HDL-c, high-density lipoprotein cholesterol; LDL-c, low-density lipoprotein cholesterol; FFA, free fatty acid; AUC, area under circle; IR, insulin resistance; LCFA, long-chain fatty acids; FATP 4, Fatty acid transporter 4; PC, Phosphatidylcholine; PE, phosphatidylethanolamine; PS, phosphatidylserine; SM, sphingolipid; Cer, ceramides; LPC, lyso-phosphatidylcholine; LPE, lyso-phosphatidylethanolamine.

## References

[1] May FJ Baer LA Lehnig AC So K Chen EY Gao F. Lipidomic Adaptations in White and Brown Adipose Tissue in Response to Exercise Demonstrate Molecular Species-Specific Remodeling. Cell Rep (2017) 18:1558–72. 10.1016/j.celrep.2017.01.038 PMC5558157 28178530

[2] Tran TT Kahn CR. Transplantation of Adipose Tissue and Stem Cells: Role in Metabolism and Disease. Nat Rev Endocrinol (2010) 6:195–213. 10.1038/nrendo.2010.20 20195269 PMC4362513

[3] Wang C Li JX Xue HF Li Y Huang JF Mai JZ. Type 2 Diabetes Mellitus Incidence in Chinese: Contributions of Overweight and Obesity. Diabetes Res Clin Pract (2015) 107:424–32. 10.1016/j.diabres.2014.09.059 25649908

[4] Nicholls DG Locke RM. Thermogenic Mechanisms in Brown Fat. Physioll Rev (1984) 64:1–64. 10.1152/physrev.1984.64.1.1 6320232

[5] Fedorenko A Lishko PV Kirichok Y. Mechanism of Fatty-Acid-Dependent UCP1 Uncoupling in Brown Fat Mitochondria. Cell (2012) 151:400–13. 10.1016/j.cell.2012.09.010 PMC3782081 23063128

[6] Carpentier AC Blondin DP Virtanen KA Richard D Haman F Turcotte ÉE. Brown Adipose Tissue Energy Metabolism in Humans. Front Endocrinol (2018) 9:447. 10.3389/fendo.2018.00447 PMC6090055 30131768

[7] De Matteis R Lucertini F Guescini M Polidori E Zeppa S Stocchi V. Exercise as a New Physiological Stimulus for Brown Adipose Tissue Activity. Nutr Metab Cardiovasc Dis (2013) 23:582–90. 10.1016/j.numecd.2012.01.013 22633794

[8] Ghorbani M Claus TH Himmshagen J. Hypertrophy of Brown Adipocytes in Brown and White Adipose Tissues and Reversal of Diet-Induced Obesity in Rats Treated With a beta3-adrenoceptor Agonist. Biochem Pharmacol (1997) 54:121–31. 10.1016/S0006-2952(97)00162-7 9296358

[9] Cypess AM Lehman S Williams G Tal I Rodman D Goldfine AB. Identification and Importance of Brown Adipose Tissue in Adult Humans. N Engl J Med (2009) 360:1509–17. 10.1056/NEJMoa0810780 PMC2859951 19357406

[10] Loyd C Obici S. Brown Fat Fuel Use and Regulation of Energy Homeostasis. Curr Opin Clin Nutr Metab Care (2014) 17:368–72. 10.1097/MCO.0000000000000063 24839950

[11] Schrauwen P van Marken Lichtenbelt WD Spiegelman BM. The Future of Brown Adipose Tissues in the Treatment of Type 2 Diabetes. Diabetologia (2015) 58:1704–07. 10.1007/s00125-015-3611-y 25957230

[12] Kaisanlahti A Glumoff T. Browning of White Fat: Agents and Implications for Beige Adipose Tissue to Type 2 Diabetes. J Physiol Biochem (2019) 75:1–10. 10.1007/s13105-018-0658-5 30506389 PMC6513802

[13] Dong X Fu J Yin X Cao S Li X Lin L. Emodin: A Review of its Pharmacology, Toxicity and Pharmacokinetics. Phytother Res (2016) 30:1207–18. 10.1002/ptr.5631 PMC7168079 27188216

[14] Heo SK Yun HJ Park WH Park SD. Emodin Inhibits TNF-α-Induced Human Aortic Smooth-Muscle Cell Proliferation Via Caspase and Mitochondrial-Dependent Apoptosis. J Cell Biochem (2008) 105:70–80. 10.1002/jcb.21805 18494000

[15] Subramaniam A Shanmugam MK Ong TH Li F Perumal E Chen L. Emodin Inhibits Growth and Induces Apoptosis in an Orthotopic Hepatocellular Carcinoma Model by Blocking Activation of STAT3. Br J Pharmacol (2013) 170:807–21. 10.1111/bph.12302 PMC3799595 23848338

[16] Zhu XF Zeng K Qiu Y Yan FH Lin CZ. Therapeutic Effect of Emodin on Collagen-Induced Arthritis in Mice. Inflammation (2013) 36:1253–9. 10.1007/s10753-013-9663-6 23729279

[17] Izhaki I. Emodin-a Secondary Metabolite With Multiple Ecological Functions in Higher Plants. New Phytol (2002) 155:205–17. 10.1046/j.1469-8137.2002.00459.x

[18] Hwang SY Heo K Kim JS Im JW Lee SM Cho M. Emodin Attenuates Radio-Resistance Induced by Hypoxia in HepG2 Cells Via the Enhancement of PARP1 Cleavage and Inhibition of JMJD2B. Oncol Rep (2015) 33:1691–8. 10.3892/or.2015.3744 25607726

[19] Tzeng TF Lu HJ Liou SS Chang CJ Liu IM. Emodin Protects Against High-Fat Diet-Induced Obesity Via Regulation of AMP-activated Protein Kinase Pathways in White Adipose Tissue. Planta Med (2012) 78:943–50. 10.1055/s-0031-1298626 22673833

[20] Song P Kim JH Ghim J Yoon JH Lee A Kwon Y. Emodin Regulates Glucose Utilization by Activating AMP-activated Protein Kinase. J Biol Chem (2013) 288:5732–42. 10.1074/jbc.M112.441477 PMC3581390 23303186

[21] Yang F Yuan PW Hao YQ Zheng ML. Emodin Enhances Osteogenesis and Inhibits Adipogenesis. BMC Complement Altern Med (2014) 14:74. 10.1186/1472-6882-14-74 24565373 PMC3974048

[22] Wang YJ Huang SL Feng Y Ning MM Leng Y. Emodin, an 11β-Hydroxysteroid Dehydrogenase Type 1 Inhibitor, Regulates Adipocyte Function In Vitro and Exerts Anti-Diabetic Effect in Ob/Ob Mice. Acta Pharmacol Sin (2012) 33:1195–203. 10.1038/aps.2012.87 PMC4003114 22922341

[23] Cheng L Dong SF Yuan YY Ma D Song JY Sun JN. Effect of Emodin on Adipose Browning in ApoE Knockout Mice. Chin J Comp Med (2018) 28:8–14. 10.3969/j.issn.1671-7856

[24] Bunyan J Murrell EA Shah PP. The Induction of Obesity in Rodents by Means of Monosodium Glutamate. Br J Nutr (1976) 35:25–39. 10.1079/BJN19760005 1106764

[25] Bernardis LL Patterson BD. Correlation Between Lee Index’ and Carcass Fat Content in Weanling and Adult Female Rats With Hypothalamic Lesions. J Endocrinol (1968) 40:527–28. 10.1677/joe.0.0400527 4868415

[26] Okla M Kim J Koehler K Chung S. Dietary Factors Promoting Brown and Beige Fat Development and Thermogenesis. Adv Nutr (2017) 8:473–83. 10.3945/an.116.014332 PMC5421122 28507012

[27] Dodd GT Decherf S Loh K Simonds SE Wiede F Balland E. Leptin and Insulin Act on POMC Neurons to Promote the Browning of White Fat. Cell (2015) 160:88–104. 10.1016/j.cell.2014.12.022 25594176 PMC4453004

[28] Ziemke F Mantzoros CS. Adiponectin in Insulin Resistance: Lessons From Translational Research. Am J Clin Nutr (2010) 91:258S–61S. 10.3945/ajcn.2009.28449C PMC2793112 19906806

[29] Lowell BB Spiegelman BM. Towards a Molecular Understanding of Adaptive Thermogenesis. Nature (2000) 404:652–60. 10.1038/35007527 10766252

[30] Artal-Sanz M Tavernarakis N. Prohibitin and Mitochondrial Biology. Trends Endocrinol Metab (2009) 20:394–401. 10.1016/j.tem.2009.04.004 19733482

[31] Feng Y Huang SL Dou W Zhang S Chen JH Shen Y. Emodin, a Natural Product, Selectively Inhibits 11b-Hydroxysteroid Dehydrogenase Type 1 and Ameliorates Metabolic Disorder in Diet-Induced Obese Mice. Br J Pharmacol (2010) 161:113–26. 10.1111/j.1476-5381.2010.00826.x PMC2962821 20718744

[32] Li J Ding L Song B Xiao X Qi M Yang QL. Emodin Improves Lipid and Glucose Metabolism in High Fat Diet-Induced Obese Mice Through Regulating SREBP Pathway. Eur J Pharmacol (2016) 770:99–109. 10.1016/j.ejphar.2015.11.045 26626587

[33] Peres Valgas da Silva C Hernández-Saavedra D White JD Stanford KI. Cold and Exercise: Therapeutic Tools to Activate Brown Adipose Tissue and Combat Obesity. Biology (2019) 8:9. 10.3390/biology8010009 PMC6466122 30759802

[34] Cavalera M Axling U Berger K Holm C. Rose Hip Supplementation Increases Energy Expenditure and Induces Browning of White Adipose Tissue. Nutr Metab (2016) 13:91. 10.1186/s12986-016-0151-5 PMC5139088 27980600

[35] Park WY Choe SK Park J Um JY. Black Raspberry (Rubus Coreanus Miquel) Promotes Browning of Preadipocytes and Inguinal White Adipose Tissue in Cold-Induced Mice. Nutrients (2019) 11:2164. 10.3390/nu11092164 PMC6769844 31509935

[36] Chen LH Chien YW Liang CT Chan CH Fan MH Huang HY. Green Tea Extract Induces Genes Related to Browning of White Adipose Tissue and Limits Weight-Gain in High Energy Diet-Fed Rat. Food Nutr Res (2017) 61:1347480. 10.1080/16546628.2017.1347480 28804438 PMC5533130

[37] Zhou L Xiao X Zhang Q Zheng J Li M Deng MQ. A Possible Mechanism: Genistein Improves Metabolism and Induces White Fat Browning Through Modulating Hypothalamic Expression of Ucn3, Depp, and Stc1. Front Endocrinol (2019) 10:478. 10.3389/fendo.2019.00478 PMC6646519 31379744

[38] Han Y Wu JZ Shen JZ Chen L He T Jin MW. Pentamethylquercetin Induces Adipose Browning and Exerts Beneficial Effects in 3T3-L1 Adipocytes and High-Fat Diet-Fed Mice. Sci Rep (2017) 7:1123. 10.1038/s41598-017-01206-4 28442748 PMC5430711

[39] Hondares E Rosell M Díaz-Delfín J Olmos Y Monsalve M Iglesias R. Peroxisome Proliferator-Activated Receptor α (Pparα) Induces Pparγ Coactivator 1α (Pgc-1α) Gene Expression and Contributes to Thermogenic Activation of Brown Fat: Involvement of PRDM16. J Biol Chem (2011) 286:43112–22. 10.1074/jbc.M111.252775 PMC3234861 22033933

[40] Wang L Teng RF Di LJ Rogers H Wu H Kopp JB. Pparα and Sirt1 Mediate Erythropoietin Action in Increasing Metabolic Activity and Browning of White Adipocytes to Protect Against Obesity and Metabolic Disorders. Diabetes (2013) 62:4122–31. 10.2337/db13-0518 PMC3837041 23990359

[41] Vessal M Mishra S Moulik S Murphy LJ. Prohibitin Attenuates Insulin-Stimulated Glucose and Fatty Acid Oxidation in Adipose Tissue by Inhibition of Pyruvate Carboxylase. FEBS J (2006) 273:568–76. 10.1111/j.1742-4658.2005.05090.x 16420480

[42] Habets DD Coumans WA Voshol PJ Boer MA Febbraio M Bonen A. AMPK-Mediated Increase in Myocardial Long-Chain Fatty Acid Uptake Critically Depends on Sarcolemmal CD36. Biochem Biophys Res Commun (2007) 355:204–10. 10.1016/j.bbrc.2007.01.141 17292863

[43] Wan Z Matravadia S Holloway GP Wright DC. FAT/CD36 Regulates PEPCK Expression in Adipose Tissue. Am J Physiol Cell Physiol (2013) 304:C478–84. 10.1152/ajpcell.00372.2012 23302781

[44] Bartelt A Bruns OT Reimer R Hohenberg H Ittrich H Peldschus K. Brown Adipose Tissue Activity Controls Triglyceride Clearance. Nat Med (2011) 17:200–5. 10.1038/nm.2297 21258337

[45] Nickerson JG Alkhateeb H Benton CR Lally J Nickerson J Han XX. Greater Transport Efficiencies of the Membrane Fatty Acid Transporters FAT/CD36 and FATP4 Compared With FABPpm and FATP1 and Differential Effects on Fatty Acid Esterification and Oxidation in Rat Skeletal Muscle. J Biol Chem (2009) 284:16522–30. 10.1074/jbc.M109.004788 PMC2713524 19380575

[46] Stahl A Gimeno RE Tartaglia LA Lodish HF. Fatty Acid Transport Proteins: A Current View of a Growing Family. Trends Endocrinol Metab (2001) 12:266–73. 10.1016/S1043-2760(01)00427-1 11445444

[47] Pol A Steven PG Parton RG. Review: Biogenesis of the Multifunctional Lipid Droplet: Lipids, Proteins, and Sites. J Cell Biol (2014) 204:635–46. 10.1083/jcb.201311051 PMC3941045 24590170

[48] Senault C Yazbeck J Goubern M Portet R Vincent M Gallay J. Relation Between Membrane Phospholipid Composition, Fluidity and Function in Mitochondria of Rat Brown Adipose Tissue. Effect of Thermal Adaptation and Essential Fatty Acid Deficiency. Biochim Biophys Acta (1990) 1023:283–9. 10.1016/0005-2736(90)90424-M 2328250

[49] Body DR. The Lipid Composition of Adipose Tissue. Prog Lipid Res (1988) 27:39–60. 10.1016/0163-7827(88)90004-5 3057509

[50] van der Veen JN Kennelly JP Wan S Vance JE Vance DE Jacobs RL. The Critical Role of Phosphatidylcholine and Phosphatidylethanolamine Metabolism in Health and Disease. Biochim Biophys Acta Biomembr (2017) 1859:1558–72. 10.1016/j.bbamem.2017.04.006 28411170

[51] Hafez IM Cullis PR. Roles of Lipid Polymorphism in Intracellular Delivery. Adv Drug Delivery Rev (2001) 47:139–48. 10.1016/S0169-409X(01)00103-X 11311989

[52] Guo Y Walther TC Rao M Stuurman N Goshima G Terayama K. Functional Genomic Screen Reveals Genes Involved in Lipid-Droplet Formation and Utilization. Nature (2008) 453:657–61. 10.1038/nature06928 PMC2734507 18408709

[53] Aitchison AJ Arsenault DJ Ridgway ND. Nuclear-Localized CTP: Phosphocholine Cytidylyltransferase α Regulates Phosphatidylcholine Synthesis Required for Lipid Droplet Biogenesis. Mol Biol Cell (2015) 26:2927–38. 10.1091/mbc.E15-03-0159 PMC4571330 26108622

[54] Petkevicius K Virtue S Bidault G Jenkins B Çubuk C Morgantini C. Accelerated Phosphatidylcholine Turnover in Macrophages Promotes Adipose Tissue Inflammation in Obesity. Elife (2019) 8:e47990. 10.7554/eLife.47990 31418690 PMC6748830

[55] Leiria LO Tseng YH. Lipidomics of Brown and White Adipose Tissue: Implications for Energy Metabolism. Biochim Biophys Acta Mol Cell Biol Lipids (2020) 1865:158788. 10.1016/j.bbalip.2020.158788 32763428 PMC7484152

